# Crystal structure, Hirshfeld surface analysis and crystal voids of 4-nitro­benzo[*c*][1,2,5]selena­diazole

**DOI:** 10.1107/S2056989024012398

**Published:** 2025-01-07

**Authors:** Atash V. Gurbanov, Tuncer Hökelek, Gunay Z. Mammadova, Khudayar I. Hasanov, Tahir A. Javadzade, Alebel N. Belay

**Affiliations:** a Excellence Center, Baku State University, Z. Xalilov Str. 23, Az 1148 Baku, Azerbaijan; b Centro de Quimica Estrutural, Instituto Superior Tecnico, Universidade de Lisboa, Av. Rovisco Pais, 1049-001 Lisbon, Portugal; c Hacettepe University, Department of Physics, 06800 Beytepe-Ankara, Türkiye; d Department of Chemistry, Baku State University, Z. Khalilov Str. 23, Az 1148 Baku, Azerbaijan; e Western Caspian University, Istiglaliyyat Str. 31, AZ 1001 Baku, Azerbaijan; f Azerbaijan Medical University, Scientific Research Centre (SRC), A. Kasumzade Str. 14, AZ 1022 Baku, Azerbaijan; g Department of Chemistry and Chemical Engineering, Khazar University, Mahzati Str. 41, AZ 1096 Baku, Azerbaijan; h Department of Chemistry, Bahir Dar University, PO Box 79, Bahir Dar, Ethiopia; University of Buenos Aires, Argentina

**Keywords:** crystal structure, non-covalent inter­actions, chalcogen bond

## Abstract

The title mol­ecule, C_6_H_3_N_3_O_2_Se, is almost planar. In the crystal, inter­molecular C—H⋯O hydrogen bonds link the mol­ecules into a network structure, enclosing *R*^2^_2_(7) and *R*^3^_3_(8) ring motifs, parallel to the *bc* plane. There are π–π inter­actions present with centroid-to-centroid distances of 3.746 (3) and 3.697 (3) Å. A Hirshfeld surface analysis of the crystal structure indicates that the most important contributions for the crystal packing are from H⋯O/O⋯H (19.6%), H⋯N/N⋯H (11.0%), H⋯Se/Se⋯H (8.5%), O⋯Se/Se⋯O (8.2%), H⋯H (7.4%), C⋯N/N⋯C (7.3%) and N⋯Se/Se⋯N (7.2%) inter­actions. Hydrogen bonding and van der Waals inter­actions are the dominant inter­actions in the crystal packing. The volume of the crystal voids and the percentage of free space were calculated to be 25.60 Å^3^ and 3.73%, showing that there is no large cavity in the crystal.

## Abstract 2

The title compound is almost planar. In the crystal, C—H⋯O hydrogen bonds link the mol­ecules into a network structure. There are also π–π inter­actions present with centroid-to-centroid distances of 3.746 (3) and 3.697 (3) Å.

## 1. Chemical context

Like other weak inter­actions, the chalcogen bond (ChB) has attracted considerable attention due to its various applications in synthesis, catalysis, crystal engineering, biochemical processes, mol­ecular recognition, functional materials, *etc*. (Mahmudov *et al.*, 2017 ▸; Mahmudov *et al.*, 2022 ▸; Scilabra *et al.*, 2019 ▸). Both bond parameters, strength and directionality of ChB can be improved by variation of substituents, ChB atom (tunability), nucleophile, resonance and cooperation of weak inter­actions (Aliyeva *et al.*, 2024 ▸; Gurbanov *et al.*, 2020 ▸). For instance, due to cooperation of the ChB, the common four-membered Se_2_N_2_ aggregate of [1,2,5]selena­diazo­les is well employed in materials chemistry (Hua *et al.*, 2020 ▸; Ho *et al.*, 2020 ▸; Tiekink, 2022 ▸). In this regard, we studied the *ortho*-NO_2_ effect on the Se_2_N_2_ synthon of 4-nitro­benzo[*c*][1,2,5]selena­diazole aggregates. We provided herein a detailed synthesis and an examination of the mol­ecular and crystal structures together with the Hirshfeld surface analysis and crystal voids of the title compound, (I).
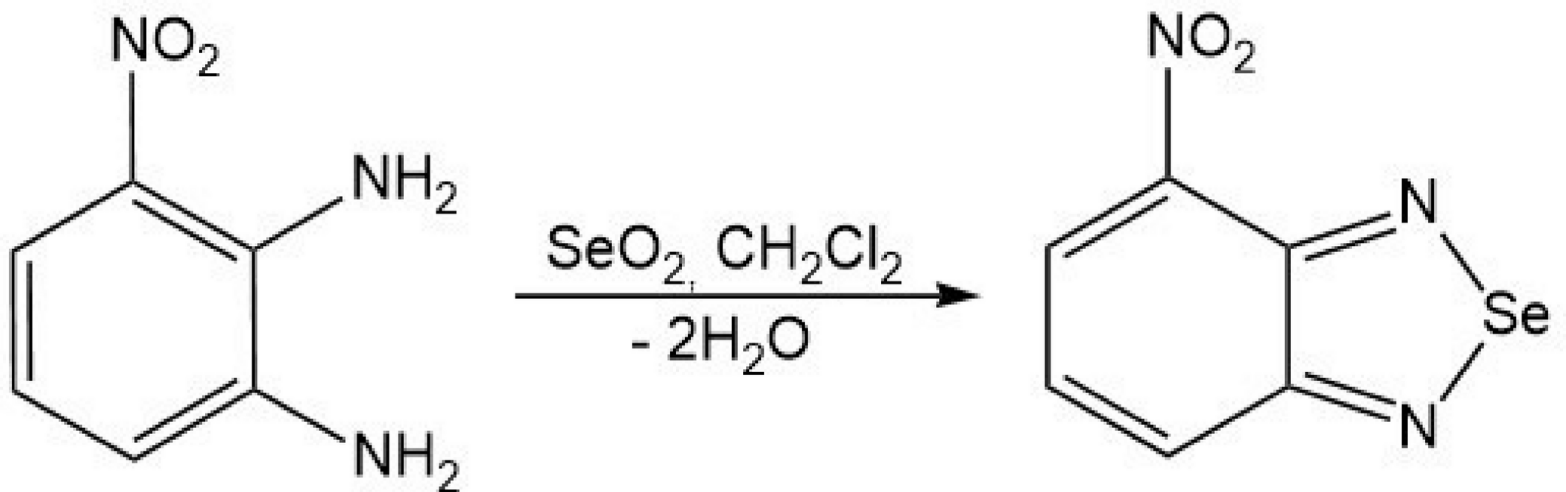


## 2. Structural commentary

**Figure 1:**
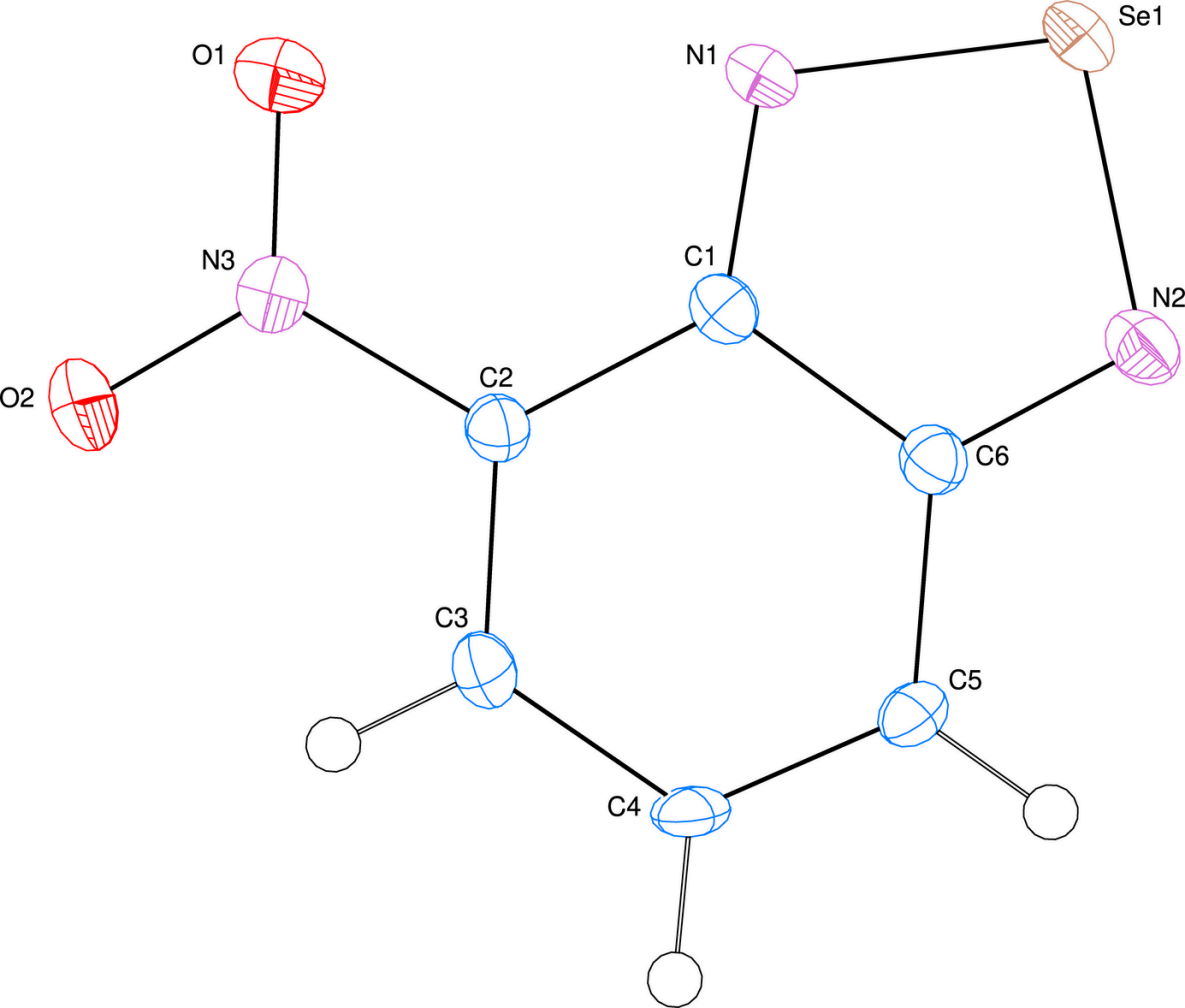
The title mol­ecule with the atom-numbering scheme and 50% probability ellipsoids.

The title compound (Fig. 1 ▸) is almost planar, with the planar *A* (C1–C6) and *B* (Se/N1/N2/C1/C6) rings oriented at a dihedral angle of *A*/*B* = 0.94 (15)°. Atoms N3, O1 and O2 are displaced by −0.004 (6), −0.024 (6) and 0.022 (6) Å, respectively, from the best least-squares plane of ring *A*. Hence, they are almost coplanar. There are no unusual bond distances or inter­bond angles in the mol­ecule.

## 3. Supra­molecular features

**Figure 2:**
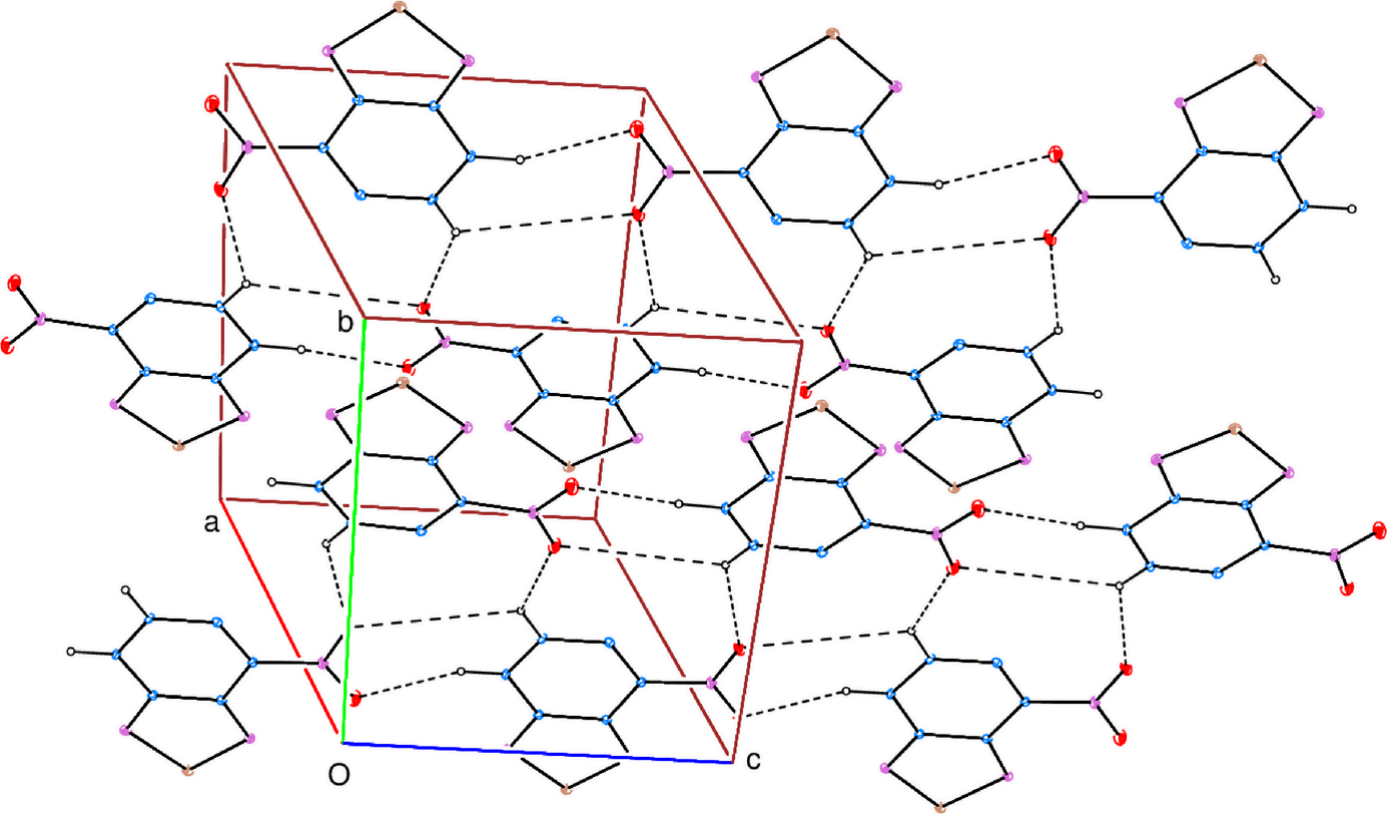
A partial packing diagram. Inter­molecular C—H⋯O hydrogen bonds are shown by dashed lines.

**Figure 3:**
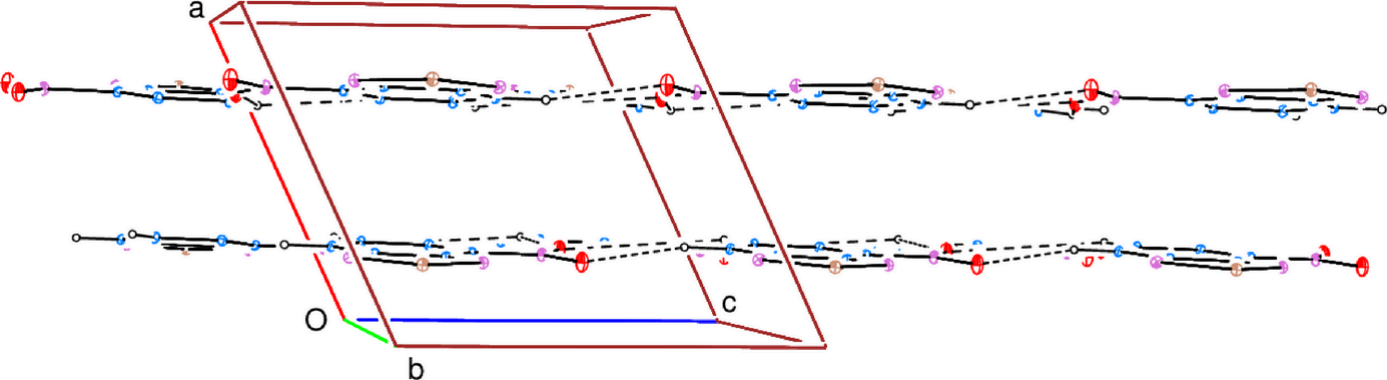
A partial packing diagram, viewed down the *b*-axis direction. Inter­molecular C—H⋯O hydrogen bonds are shown by dashed lines.

In the crystal, inter­molecular C—H⋯O hydrogen bonds (Table 1 ▸) link the mol­ecules into a network structure, enclosing 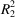
(7) and 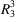
(8) ring motifs (Fig. 2 ▸), parallel to the *bc* plane (Fig. 3 ▸). No C—H⋯π(ring) inter­actions are observed but there are two π–π inter­actions between the almost parallel *A* and *B* rings and also between the parallel *B* rings with centroid-to-centroid distances of 3.746 (3) and 3.697 (3) Å, respectively [*Cg*1⋯*Cg*2^i^ = 3.746 (3) Å with α = 0.91° and *Cg*2⋯*Cg*2^i^ = 3.697 (3) Å with α = 0.00° where *Cg*1 and *Cg*2 are the centroids of rings *A* and *B*, respectively; symmetry code: (i) −*x*, −*y*, 1 − *z*].

## 4. Hirshfeld surface analysis

**Figure 4:**
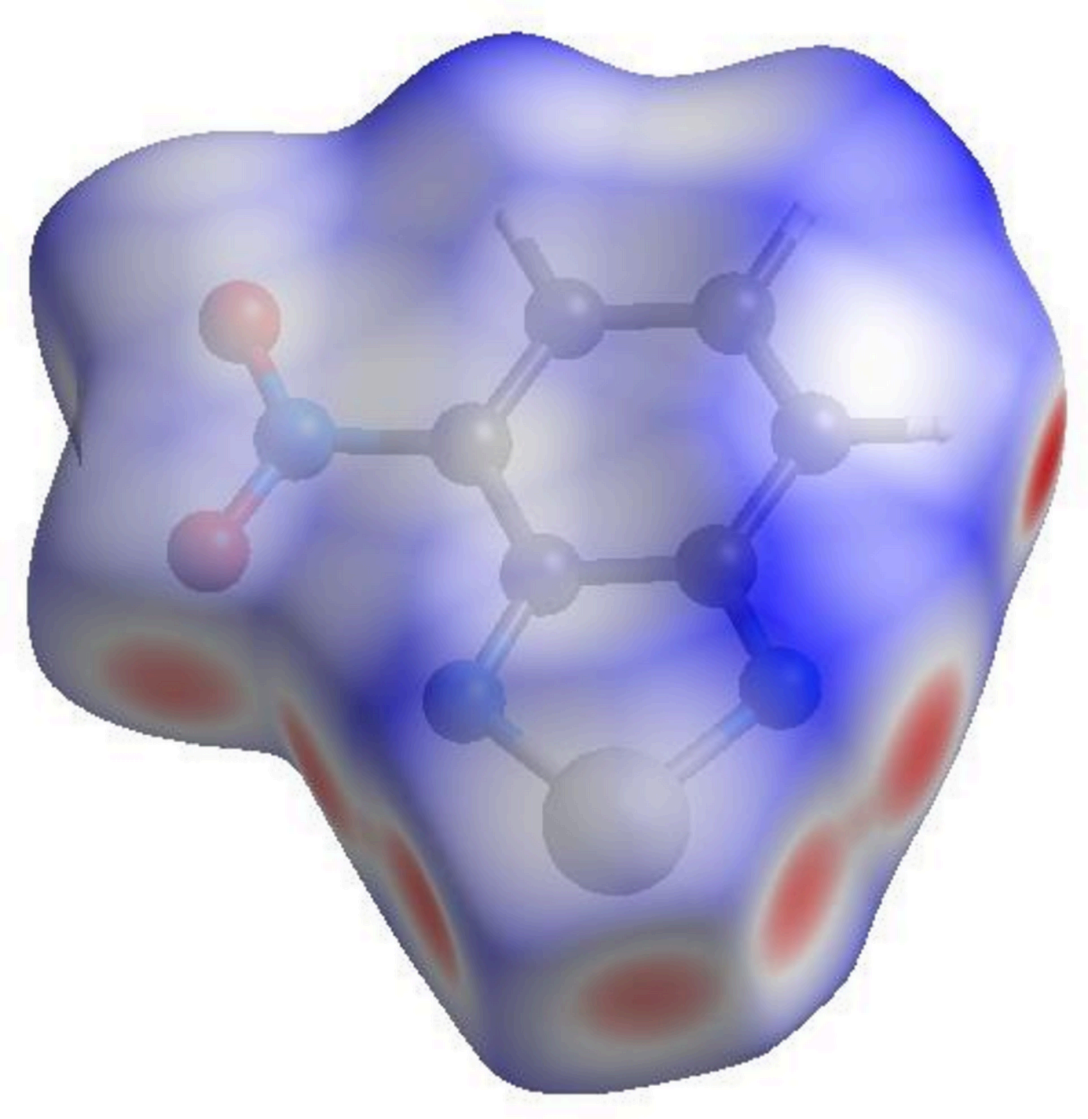
View of the three-dimensional Hirshfeld surface of the title compound plotted over *d*_norm_.

**Figure 5:**
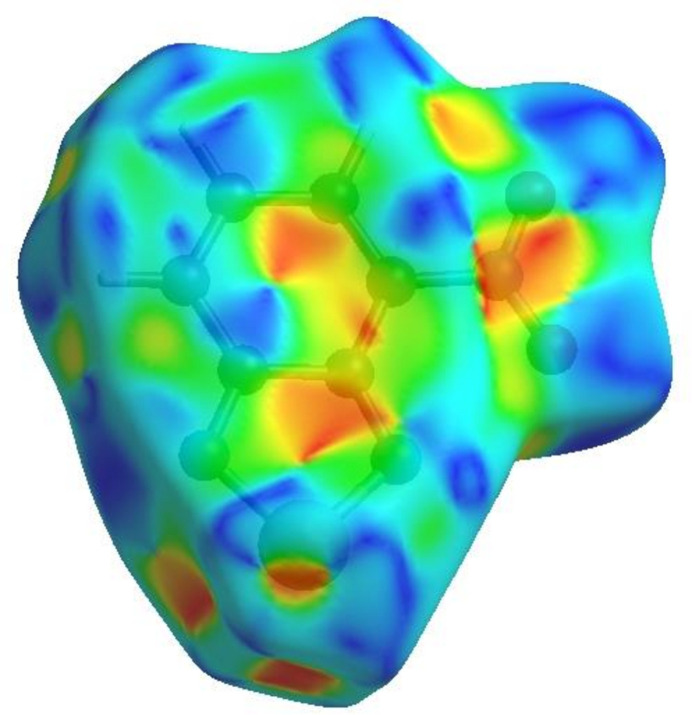
Hirshfeld surface of the title compound plotted over shape-index.

**Figure 6:**
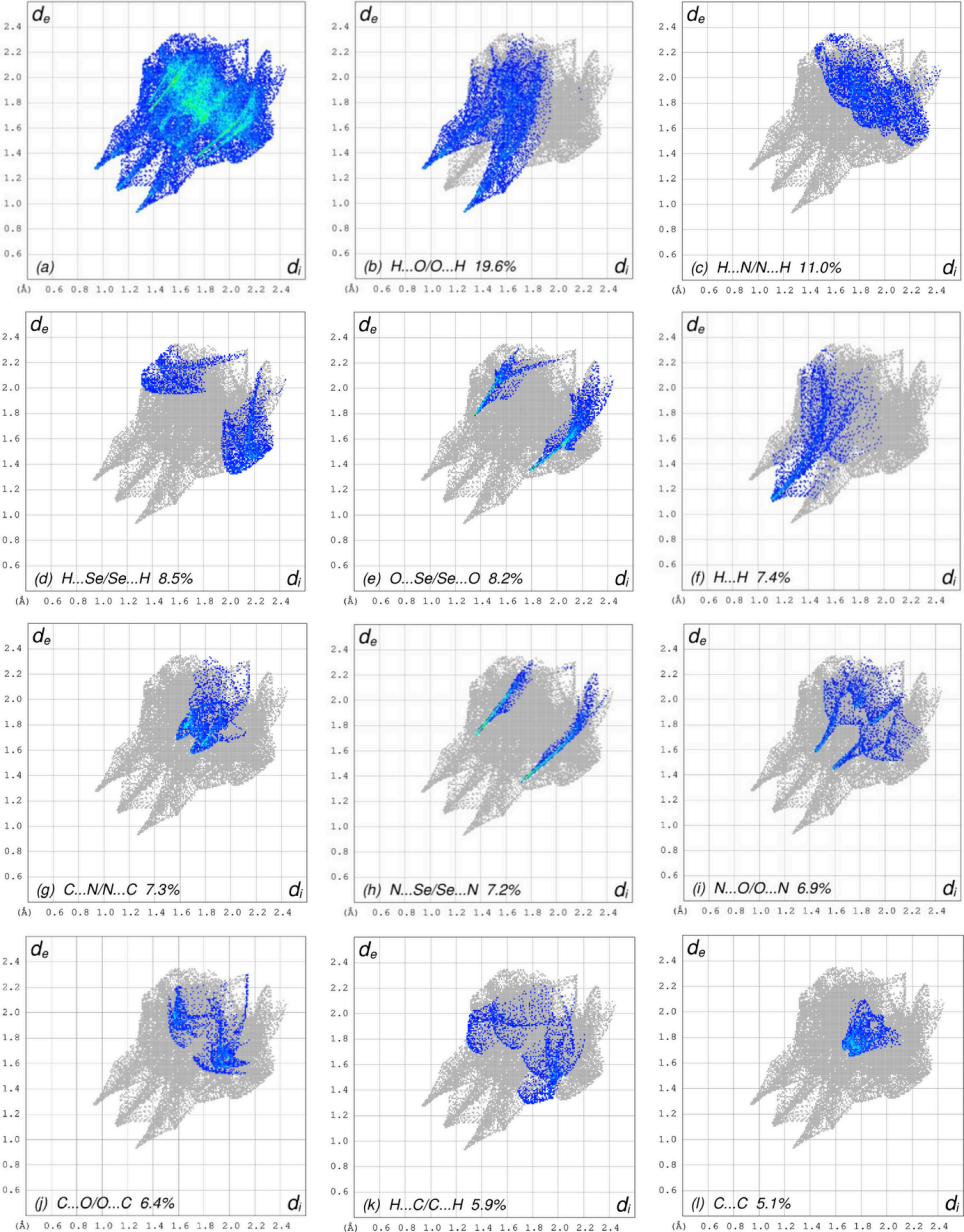
The full two-dimensional fingerprint plots for the title compound, showing (*a*) all inter­actions, and delineated into (*b*) H⋯O/O⋯H, (*c*) H⋯N/N⋯H, (*d*) H⋯Se/Se⋯H, (*e*) O⋯Se/Se⋯O, (*f*) H⋯H, (*g*) C⋯N/N⋯C, (*h*) N⋯Se/Se⋯N, (*i*) N⋯O/O⋯N, (*j*) C⋯O/O⋯C, (*k*) H⋯C/C ⋯H, (*l*) C⋯C, (*m*) N⋯N, (*n*) O⋯O and (*o*) C⋯Se/Se⋯C inter­actions. The *d*_i_ and *d*_e_ values are the closest inter­nal and external distances (in Å) from given points on the Hirshfeld surface.

In order to visualize the inter­molecular inter­actions, a Hirshfeld surface (HS) analysis (Hirshfeld, 1977 ▸; Spackman & Jayatilaka, 2009 ▸) was carried out using *Crystal Explorer 17.5* (Spackman *et al.*, 2021 ▸). In the HS plotted over *d*_norm_ (Fig. 4 ▸), the white areas indicate contacts with distances equal to the sum of van der Waals radii, and the red and blue colours indicate distances shorter (in close contact) or longer (distinct contact) than the van der Waals radii, respectively (Venkatesan *et al.*, 2016 ▸). The bright-red spots indicate their roles as the respective donors and/or acceptors. The shape-index surface can be used to identify characteristic packing modes, in particular, planar stacking arrangements and the presence of aromatic stacking inter­actions such as C—H⋯π and π–π inter­actions. C—H⋯π inter­actions are represented as red *p*-holes, which are related to the electron ring inter­actions between the CH groups and the centroid of the aromatic rings of neighbouring mol­ecules. Fig. 5 ▸ clearly suggests that there are no C—H⋯π inter­actions in (I). The shape-index is a tool for visualizing π–π stacking by the presence of adjacent red and blue triangles; if there are no adjacent red and/or blue triangles, then there are no π–π inter­actions. Fig. 5 ▸ clearly suggests that there are π–π inter­actions in (I). The overall two-dimensional fingerprint plot, Fig. 6 ▸*a*, and those delineated into H⋯O/O⋯H, H⋯N/N⋯H, H⋯Se/Se⋯H, O⋯Se/Se⋯O, H⋯H, C⋯N/N⋯C, N⋯Se/Se⋯N, N⋯O/O⋯N, C⋯O/O⋯C, H⋯C/C⋯H, C⋯C, N⋯N, O⋯O and C⋯Se/Se⋯C (McKinnon *et al.*, 2007 ▸) are illustrated in Fig. 6 ▸*b*–*o*, respectively, together with their relative contributions to the Hirshfeld surface. The most important inter­action is H⋯O/O⋯H (Table 2 ▸) contributing 19.6% to the overall crystal packing, which is reflected in Fig. 6 ▸*b* as a pair of spikes with the tips at *d*_e_ + *d*_i_ = 2.20 Å. The H⋯N/N⋯H contacts (Fig. 6 ▸*c*) make an 11.0% contribution to the HS and have the tips at *d*_e_ + *d*_i_ = 3.46 Å. The H⋯Se/Se⋯H contacts (Fig. 6 ▸*d*; 8.5% contribution to the HS) have a pair of wings with the tips at *d*_e_ + *d*_i_ = 3.34 Å. The pair of spikes for the O⋯Se/Se⋯O contacts (Table 2 ▸ and Fig. 6 ▸*e*), contributing 8.2% to the HS, have the tips at *d*_e_ + *d*_i_ = 3.14 Å. The H⋯H inter­actions (Fig. 6 ▸*f*) contribute 7.4% to the HS with the tip at *d*_e_ = *d_i_* = 1.12 Å. The C⋯N/N⋯C (Fig. 6 ▸*g*), N⋯Se/Se⋯N (Table 2 ▸ and Fig. 6 ▸*h*) and N⋯O/O ⋯N (Table 2 ▸ and Fig. 6 ▸*i*) contacts contribute 7.3%, 7.2% and 6.9%, respectively, to the HS and are viewed as pairs of spikes with the tips at *d*_e_ + *d*_i_ = 3.26, 3.08 and 3.04 Å, respectively. The C⋯O/ O⋯C contacts (Fig. 6 ▸*j*) make 6.4% contribution to the HS with the central point at *d*_e_ = *d*_i_ = 1.72 Å. In the absence of C—H⋯π inter­actions, the H⋯C/C⋯H contacts, contributing 5.9% to the overall crystal packing, are reflected in Fig. 6 ▸*k* with the tips at *d*_e_ + *d*_i_ = 3.46 Å. The C⋯C contacts (Fig. 6 ▸*l*) contributing 5.1% to the HS have a bullet-shaped distribution of points with the tip at *d*_e_ = *d*_i_ = 1.69Å. Finally, the N⋯N (Table 2 ▸ and Fig. 6 ▸*m*), O⋯O (Fig. 6 ▸*n*) and C⋯Se/Se⋯C (Fig. 6 ▸*o*) contacts with 3.3%, 2.1% and 1.1% contributions, respectively, to the HS have very low densities.

**Figure 7:**
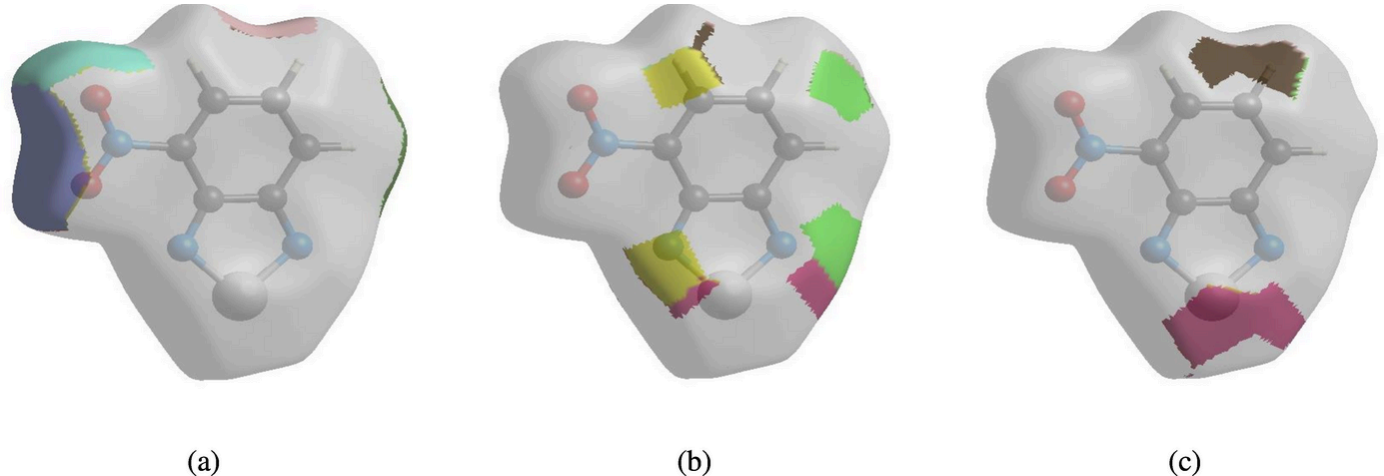
The Hirshfeld surface representations with the function *d*_norm_ plotted onto the surface for (*a*) H⋯O/O⋯H, (*b*) H⋯N/N⋯H and (*c*) H⋯Se/Se⋯H inter­actions.

The nearest neighbour coordination environment of a mol­ecule can be determined from the colour patches on the HS based on how close to other mol­ecules they are. The Hirshfeld surface representations with the function *d*_norm_ plotted onto the surface are shown for the H⋯O/O⋯H, H⋯N/N⋯H and H⋯Se/Se⋯H inter­actions in Fig. 7 ▸*a*–*c*, respectively.

The Hirshfeld surface analysis confirms the importance of H-atom contacts in establishing the packing. The large number of H⋯O/O⋯H, H⋯N/N⋯H and H⋯Se/Se⋯H inter­actions suggest that van der Waals inter­actions and hydrogen bonding play the major roles in the crystal packing (Hathwar *et al.*, 2015 ▸).

## 5. Crystal voids

**Figure 8:**
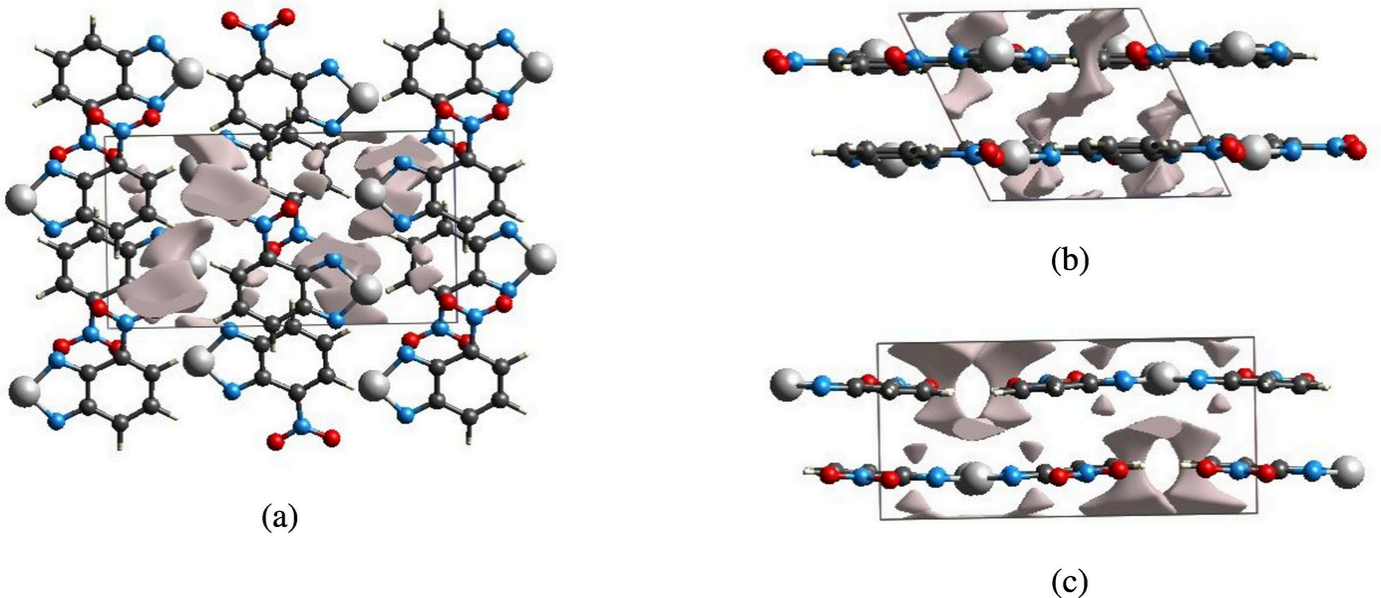
Graphical views of voids in the crystal packing of (I) (*a*) along the *a*-axis direction, (*b*) along the *b*-axis direction and (*c*) along the *c*-axis direction.

The strength of the crystal packing is important for determining the response to an applied mechanical force. If the crystal packing results in significant voids, the mol­ecules are not tightly packed and a small amount of applied external mechanical force may easily break the crystal. To check the mechanical stability of the crystal, a void analysis was performed by adding up the electron densities of the spherically symmetric atoms contained in the asymmetric unit (Turner *et al.*, 2011 ▸). The void surface is defined as an isosurface of the procrystal electron density and is calculated for the whole unit cell where the void surface meets the boundary of the unit cell and capping faces are generated to create an enclosed volume. The volume of the crystal voids (Fig. 8 ▸*a*–*c*) and the percentage of free space in the unit cell are calculated as 25.60 Å^3^ and 3.73%, respectively. Thus, the crystal packing appears compact and the mechanical stability should be substantial.

## 6. Database survey

A survey conducted of the Cambridge Structural Database (CSD, Version 5.45, last updated September 2024; Groom *et al.*, 2016 ▸) indicates that two mol­ecules are similar to the title compound (I): (*rac*)-4-methyl-4-nitro-2,1,3-benzoselena-diazol-5(4*H*)-one, C_7_H_5_N_3_O_3_Se (CSD refcode JURLAJ; Tian *et al.*, 1993 ▸) and 5-nitro-2,1,3-benzoselena­diazole, C_6_H_3_N_3_O_2_Se (CSD refcode DOBWUQ; Aliyeva *et al.*, 2023 ▸).

## 7. Synthesis and crystallization

3-Nitro­benzene-1,2-di­amine (10 mmol) and selenium dioxide (10 mmol) were dissolved in 25 ml of di­chloro­methane and stirred for 1 h at ambient temperature, and further refluxed for 1 h (Georges *et al.*, 2024 ▸). After cooling to room temperature, the solvent was evaporated under reduced pressure to give the reaction product. Crystals suitable for X-ray analysis were obtained by slow evaporation of a methanol solution. Yield 82% (based on SeO_2_), yellow powder soluble in methanol, ethanol and DMSO. Analysis calculated for C_6_H_3_N_3_O_2_Se (*M*_r_ = 228.07): C, 31.60; H, 1.33; N, 18.42. Found: C, 31.58, H, 1.30; N, 18.40. ESI–MS (positive ion mode), *m*/*z*: 229.10 [*M*_r_ + H]^+. 1^H NMR (DMSO-*d*^6^), δ: 7.72–8.46 (3H, Ar-H). ^13^C NMR (DMSO-*d*^6^), 126.4, 126.8, 129.4, 140.5, 149.9 and 159.9.

## 8. Refinement

Crystal data, data collection and structure refinement details are summarized in Table 3 ▸. The C-bond H atoms were positioned geometrically (C—H = 0.95 Å) and refined using a riding model with *U*_iso_(H) = 1.2*U*_eq_(C).

## Supplementary Material

Crystal structure: contains datablock(s) I. DOI: 10.1107/S2056989024012398/vu2008sup1.cif

Structure factors: contains datablock(s) I. DOI: 10.1107/S2056989024012398/vu2008Isup2.hkl

Supporting information file. DOI: 10.1107/S2056989024012398/vu2008Isup3.cml

CCDC reference: 2412565

Additional supporting information:  crystallographic information; 3D view; checkCIF report

## Figures and Tables

**Table 1:** Hydrogen-bond geometry (Å, °)

*D*—H⋯*A*	*D*—H	H⋯*A*	*D*⋯*A*	*D*—H⋯*A*
C4—H4⋯O2^iv^	0.95	2.52	3.269 (7)	135
C5—H5⋯O1^iii^	0.95	2.33	3.240 (7)	161

Symmetry codes: (iii) 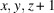; (iv) 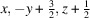.

**Table 2:** Selected interatomic distances (Å)

Se1⋯O1^i^	3.140 (4)	O2⋯H3	2.37
Se1⋯N1^i^	3.132 (5)	H4⋯O2^iv^	2.52
Se1⋯N2^ii^	3.079 (5)	N1⋯N3	3.012 (6)
O1⋯N1	2.709 (6)	N1⋯N2^ii^	3.017 (6)
H5⋯O1^iii^	2.33		

Symmetry codes: (i) 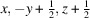; (ii) 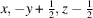; (iii) 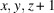; (iv) 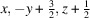.

**Table 3:** Experimental details

Crystal data	
Chemical formula	C_6_H_3_N_3_O_2_Se
*M*_r_	228.07
Crystal system, space group	Monoclinic, *P*2_1_/*c*
Temperature (K)	150
*a*, *b*, *c* (Å)	7.0105 (4), 13.2765 (8), 8.1311 (5)
β (°)	114.808 (3)
*V* (Å^3^)	686.96 (7)
*Z*	4
Radiation type	Mo *K*α
μ (mm^−1^)	5.42
Crystal size (mm)	0.28 × 0.21 × 0.14

Data collection	
Diffractometer	Bruker APEXII CCD
Absorption correction	Multi-scan (*SADABS*; Krause *et al.*, 2015 ▸)
*T*_min_, *T*_max_	0.284, 0.473
No. of measured, independent and observed [*I* > 2σ(*I*)] reflections	5894, 1475, 1297
*R*_int_	0.037
(sin θ/λ)_max_ (Å^−1^)	0.636

Refinement	
*R*[*F*^2^ > 2σ(*F*^2^)], *wR*(*F*^2^), *S*	0.044, 0.104, 1.17
No. of reflections	1475
No. of parameters	109
H-atom treatment	H-atom parameters constrained
Δρ_max_, Δρ_min_ (e Å^−3^)	1.24, −1.27

Computer programs: *APEX4* and *SAINT* (Bruker, 2014), *SHELXT2019/1* (Sheldrick, 2015*a* ▸), *SHELXL2019/1* (Sheldrick, 2015*b* ▸) and *SHELXTL* (Sheldrick, 2008 ▸).

## supplementary crystallographic information

### 4-Nitrobenzo[c][1,2,5]selenadiazole Crystal data

**Table:**

C_6_H_3_N_3_O_2_Se	*F*(000) = 440
*M**_r_* = 228.07	*D*_x_ = 2.205 Mg m^−^^3^
Monoclinic, *P*2_1_/*c*	Mo *K*α radiation, λ = 0.71073 Å
*a* = 7.0105 (4) Å	Cell parameters from 2556 reflections
*b* = 13.2765 (8) Å	θ = 3.1–26.7°
*c* = 8.1311 (5) Å	µ = 5.42 mm^−^^1^
β = 114.808 (3)°	*T* = 150 K
*V* = 686.96 (7) Å^3^	Prism, yellow
*Z* = 4	0.28 × 0.21 × 0.14 mm

### 4-Nitrobenzo[c][1,2,5]selenadiazole Data collection

**Table:**

Bruker APEXII CCD diffractometer	1297 reflections with *I* > 2σ(*I*)
φ and ω scans	*R*_int_ = 0.037
Absorption correction: multi-scan (SADABS; Krause *et al.*, 2015)	θ_max_ = 26.9°, θ_min_ = 3.1°
*T*_min_ = 0.284, *T*_max_ = 0.473	*h* = −8→8
5894 measured reflections	*k* = −16→16
1475 independent reflections	*l* = −10→10

### 4-Nitrobenzo[c][1,2,5]selenadiazole Refinement

**Table:**

Refinement on *F*^2^	Primary atom site location: structure-invariant direct methods
Least-squares matrix: full	Secondary atom site location: difference Fourier map
*R*[*F*^2^ > 2σ(*F*^2^)] = 0.044	Hydrogen site location: inferred from neighbouring sites
*wR*(*F*^2^) = 0.104	H-atom parameters constrained
*S* = 1.17	*w* = 1/[σ^2^(*F*_o_^2^) + 6.1884*P*] where *P* = (*F*_o_^2^ + 2*F*_c_^2^)/3
1475 reflections	(Δ/σ)_max_ < 0.001
109 parameters	Δρ_max_ = 1.24 e Å^−^^3^
0 restraints	Δρ_min_ = −1.27 e Å^−^^3^

### 4-Nitrobenzo[c][1,2,5]selenadiazole Special details

**Table:**

Geometry. All esds (except the esd in the dihedral angle between two l.s. planes) are estimated using the full covariance matrix. The cell esds are taken into account individually in the estimation of esds in distances, angles and torsion angles; correlations between esds in cell parameters are only used when they are defined by crystal symmetry. An approximate (isotropic) treatment of cell esds is used for estimating esds involving l.s. planes.

### 4-Nitrobenzo[c][1,2,5]selenadiazole Fractional atomic coordinates and isotropic or equivalent isotropic displacement parameters (Å^2^)

**Table:**

	*x*	*y*	*z*	*U*_iso_*/*U*_eq_	
Se1	0.77441 (9)	0.25835 (4)	0.82143 (8)	0.01947 (18)	
O1	0.7737 (8)	0.4739 (3)	0.3944 (6)	0.0372 (11)	
O2	0.7408 (8)	0.6348 (3)	0.4079 (6)	0.0324 (11)	
N1	0.7713 (7)	0.3520 (3)	0.6607 (6)	0.0179 (10)	
N2	0.7564 (7)	0.3476 (3)	0.9783 (6)	0.0198 (10)	
N3	0.7548 (8)	0.5496 (3)	0.4731 (6)	0.0219 (10)	
C1	0.7570 (8)	0.4405 (4)	0.7304 (7)	0.0152 (10)	
C2	0.7477 (8)	0.5380 (4)	0.6503 (7)	0.0155 (10)	
C3	0.7288 (8)	0.6233 (4)	0.7359 (7)	0.0175 (11)	
H3	0.720824	0.686805	0.679574	0.021*	
C4	0.7207 (8)	0.6192 (4)	0.9056 (7)	0.0174 (11)	
H4	0.707675	0.680103	0.961342	0.021*	
C5	0.7310 (8)	0.5300 (4)	0.9926 (7)	0.0164 (10)	
H5	0.726556	0.528392	1.107708	0.020*	
C6	0.7487 (8)	0.4387 (4)	0.9056 (7)	0.0161 (10)	

### 4-Nitrobenzo[c][1,2,5]selenadiazole Atomic displacement parameters (Å^2^)

**Table:**

	*U*^11^	*U*^22^	*U*^33^	*U*^12^	*U*^13^	*U*^23^
Se1	0.0249 (3)	0.0107 (2)	0.0233 (3)	0.0001 (2)	0.0106 (2)	0.0017 (2)
O1	0.066 (3)	0.019 (2)	0.034 (3)	0.001 (2)	0.029 (2)	−0.0030 (18)
O2	0.054 (3)	0.019 (2)	0.029 (2)	0.0017 (19)	0.022 (2)	0.0081 (17)
N1	0.023 (3)	0.012 (2)	0.020 (2)	0.0017 (17)	0.009 (2)	−0.0001 (17)
N2	0.022 (2)	0.014 (2)	0.024 (3)	0.0000 (17)	0.011 (2)	0.0018 (18)
N3	0.028 (3)	0.020 (2)	0.020 (3)	0.0003 (19)	0.012 (2)	0.0011 (19)
C1	0.013 (2)	0.014 (2)	0.018 (3)	−0.0010 (18)	0.006 (2)	0.0004 (19)
C2	0.017 (3)	0.015 (2)	0.017 (3)	0.0001 (19)	0.008 (2)	0.0010 (19)
C3	0.019 (3)	0.015 (2)	0.017 (3)	0.001 (2)	0.006 (2)	0.003 (2)
C4	0.022 (3)	0.014 (2)	0.019 (3)	0.001 (2)	0.012 (2)	−0.0055 (19)
C5	0.019 (3)	0.018 (2)	0.015 (3)	0.001 (2)	0.010 (2)	−0.002 (2)
C6	0.016 (3)	0.015 (2)	0.018 (3)	0.0005 (19)	0.009 (2)	0.0011 (19)

### 4-Nitrobenzo[c][1,2,5]selenadiazole Geometric parameters (Å, º)

**Table:**

Se1—N2	1.784 (5)	C1—C6	1.450 (7)
Se1—N1	1.797 (4)	C2—C3	1.364 (7)
O1—N3	1.228 (6)	C3—C4	1.406 (7)
O2—N3	1.235 (6)	C3—H3	0.9500
N1—C1	1.327 (6)	C4—C5	1.366 (7)
N2—C6	1.338 (7)	C4—H4	0.9500
N3—C2	1.471 (7)	C5—C6	1.434 (7)
C1—C2	1.439 (7)	C5—H5	0.9500
			
Se1···O1^i^	3.140 (4)	O2···H3	2.37
Se1···N1^i^	3.132 (5)	H4···O2^iv^	2.52
Se1···N2^ii^	3.079 (5)	N1···N3	3.012 (6)
O1···N1	2.709 (6)	N1···N2^ii^	3.017 (6)
H5···O1^iii^	2.33		
			
N2—Se1—N1	94.46 (19)	C2—C3—C4	121.4 (5)
C1—N1—Se1	106.4 (3)	C2—C3—H3	119.3
C6—N2—Se1	106.7 (4)	C4—C3—H3	119.3
O1—N3—O2	122.2 (5)	C5—C4—C3	121.8 (5)
O1—N3—C2	118.7 (4)	C5—C4—H4	119.1
O2—N3—C2	119.1 (4)	C3—C4—H4	119.1
N1—C1—C2	126.9 (5)	C4—C5—C6	118.3 (5)
N1—C1—C6	116.5 (5)	C4—C5—H5	120.8
C2—C1—C6	116.5 (4)	C6—C5—H5	120.8
C3—C2—C1	120.7 (5)	N2—C6—C5	122.8 (5)
C3—C2—N3	117.6 (4)	N2—C6—C1	116.0 (5)
C1—C2—N3	121.6 (4)	C5—C6—C1	121.2 (5)
			
N2—Se1—N1—C1	−0.2 (4)	C1—C2—C3—C4	0.9 (8)
N1—Se1—N2—C6	0.2 (4)	N3—C2—C3—C4	180.0 (5)
Se1—N1—C1—C2	−179.5 (4)	C2—C3—C4—C5	−0.1 (9)
Se1—N1—C1—C6	0.2 (6)	C3—C4—C5—C6	−0.5 (8)
N1—C1—C2—C3	178.7 (5)	Se1—N2—C6—C5	179.2 (4)
C6—C1—C2—C3	−1.0 (7)	Se1—N2—C6—C1	−0.1 (6)
N1—C1—C2—N3	−0.3 (8)	C4—C5—C6—N2	−178.9 (5)
C6—C1—C2—N3	180.0 (5)	C4—C5—C6—C1	0.4 (8)
O1—N3—C2—C3	179.4 (5)	N1—C1—C6—N2	0.0 (7)
O2—N3—C2—C3	−0.9 (8)	C2—C1—C6—N2	179.7 (5)
O1—N3—C2—C1	−1.6 (8)	N1—C1—C6—C5	−179.4 (5)
O2—N3—C2—C1	178.1 (5)	C2—C1—C6—C5	0.4 (7)

Symmetry codes: (i) *x*, −*y*+1/2, *z*+1/2; (ii) *x*, −*y*+1/2, *z*−1/2; (iii) *x*, *y*, *z*+1; (iv) *x*, −*y*+3/2, *z*+1/2.

### 4-Nitrobenzo[c][1,2,5]selenadiazole Hydrogen-bond geometry (Å, º)

**Table:**

*D*—H···*A*	*D*—H	H···*A*	*D*···*A*	*D*—H···*A*
C4—H4···O2^iv^	0.95	2.52	3.269 (7)	135
C5—H5···O1^iii^	0.95	2.33	3.240 (7)	161

Symmetry codes: (iii) *x*, *y*, *z*+1; (iv) *x*, −*y*+3/2, *z*+1/2.
